# Virtual Reality and Wearable Technologies to Support Adaptive Responding of Children and Adolescents With Neurodevelopmental Disorders: A Critical Comment and New Perspectives

**DOI:** 10.3389/fpsyg.2021.720626

**Published:** 2021-07-12

**Authors:** Fabrizio Stasolla

**Affiliations:** Faculty of Law, Giustino Fortunato University, Benevento, Italy

**Keywords:** assistive technology, virtual reality, wearable technologies, assessment, rehabilitation, quality of life, neurodevelopmental disorders

## Introduction

Children and adolescents with neurodevelopmental disorders (NDD) are commonly described as cognitively, socially, emotionally, and communicatively impaired. Usually, attention deficits hyperactivity disorders (ADHD), autism spectrum disorders (ASD), and cerebral palsy (CP) are included. Furthermore, learning difficulties are observed, and academic failures are documented (Rey-Casserly et al., 2019). Additionally, their chronic clinical conditions may represent a relevant burden for both caregivers and families, next to a significant threat for their health and a cost for medical services (Hickie et al., 2019; Kim et al., 2019). Covid-19 pandemic dramatically and suddenly exacerbated their fragile health conditions due to unavailability of medical or rehabilitative services and preventive measures such as quarantine and social distancing (Ehrler et al., 2021; Jaffee, 2021; Masi et al., 2021). In fact, their quality of life may be seriously hampered and both caregivers and families's burden may drastically increase (Dhiman et al., 2020; Townsend, 2020; Farajzadeh et al., 2021; Zimmer et al., 2021). Technology-aided options provide the opportunity to independently administrate and self-regulate useful interventions for both assessment and rehabilitative purposes (Rahman et al., 2020). Beside assistive technology-based programs (Stasolla and Bottiroli, 2020), one may envisage the use of virtual reality setups (VR) and/or wearable technologies (WT) (Vona et al., 2020).

VR offers several advantages as ecological validity, experimental control, and behavioral responses tracking (Yaremych and Persky, 2019). Wearable technologies may be easily transportable and may represent a valid option for monitoring and recovery of an adaptive responding (Gorman et al., 2002). A concise review of the literature available along the last five years (i.e., 2017–2021) was carried out. Ten contributions were critically discussed (i.e., five on VR and five on WT, see Table 1). Although ADHD and ASD populations were widely targeted, cerebral palsy and rare genetic diseases were still uninvestigated (Valentine et al., 2020). Moreover, communication, emotional, social skills, and posture were evaluated (Berenguer et al., 2020). Conversely, challenging behaviors, positive occupation (e.g., request and choice capacities), and leisure options were relatively ignored (Airaksinen et al., 2020).

**Table 1:** Reviewed studies arranged in alphabetic order.

**References**	**Population**	**Targeted Behaviors**	**Group**	**Main Outcomes**
Airaksinen et al. (2020)	ASD	Posture	WT	Device effectiveness
Billeci et al. (2018)	ASD	Joint attention	WT	Protocol study
Berenguer et al. (2020)	ASD	Well-being	VR	Technology suitability
Colombini et al. (2021)	ADHD + ASD	Visual interactions	VR	Setup affordability
Dellazizzo et al. (2020)	ADHD + ADHD	Cognitive functioning	VR	Low evidence quality
Di Palma et al. (2017)	ASD	Socio-cognitive tasks	WT	Device efficacy
Gualniera et al. (2021)	RTT	Emotional dysregulation	WT	Technology suitability
Romero-Ayuso et al. (2020)	ADHD + ASD	Self-regulation	VR	Protocol study
Sgandurra et al. (2018)	CP	Motor movements	WT	Protocol study
Wiguna et al. (2020)	ADHD	Executive functions	VR	Setup reliability

Along last decade, evidence-based research on the use of digital tools to promote adaptive skills and communication opportunities in individuals with neurodevelopmental disabilities meaningfully increased (Lussier-Desrochers et al., 2020). An illustrative example is represented by Operto et al. (2020) who emphasized that hat a longer time of exposure to digital tools was related to lower mimic-gestural skills in children from 8–17 months and to lower language skills in children between 18 and 36 months, regardless of age, gender, socio-economic status, content, and modality of use.

A critical comment on the literature available within this framework was detailed and constructively argued. A new home-based cognitive rehabilitation software recently proposed in patients with neurodegenerative diseases and a virtual reality setup as a technological-aided strategy (Bernini et al., 2021; Stasolla et al., 2021) were presented as a perspective solution during Covid-19 pandemic era. Thus, social distancing and quarantine preventive measures forced and confined children and adolescents with NDD in their homes. Professionals and clinicians may remotely supervise and evaluate their patients through telerehabilitation strategies (Caprì et al., 2021; Varela-Aldas et al., 2021).

## Virtual Reality

VR, including augmented reality (AR), recently emerged as crucial means of suitable intervention in different area of public health, namely (a) assessment, (b) diagnosis, (c) recovery, and (d) well-being. With regard to rehabilitative program, VR has been widely used to favorably address NDD such as attention deficits hyperactivity disorders (ADHD) and autism spectrum disorders (ASD). VR ensures individuals with NDD with sensory experiences, computer-mediated in artificial environments, enhancing virtual interactions similarly to the real life. AR, as part of VR, enables an interaction in a physical condition, differently from the artificial context provided by VR. That is, VR usually requires the use of specific headsets, which may not be easily wearable for individuals with NDD. Conversely, AR may be considered as simpler because it refers to smartphones, tablets, and I-PAD, which are more adaptable to the real world (Liu et al., 2017; Mesa-Gresa et al., 2018; Akin and Gokturk, 2019).

Berenguer et al. (2020) systematically reviewed the literature on this specific topic among children and adolescents with ASD and emphasized the effectiveness of AR-based programs to promote and support health, well-being, and quality of life in individuals with ASD. Wiguna et al. (2020) proposed a four-step method as a mixed method research design aimed at combining qualitative and quantitative approaches to reduce bias and collect reliable data. An ADHD-VR digital game diagnostic-tool prototype with a deep learning application for children was successfully developed. Dellazizzo et al. (2020) carried out a meta-analysis and summarized the current state of evidence on VR-based interventions for psychiatric disorders by assessing the quality of feasibility and suitability evidenced by the meta-analysis itself. Results demonstrated that the quality of evidence varied between low and moderate. Lower quality evidence was explained by limited number of randomized controlled trials, lack of follow-up and control groups, the heterogeneity and publication bias.

Romero-Ayuso et al. (2020) assessed a protocol-study to evaluate self-regulation ability in children with NDD (i.e., specifically ADHD and ASD participants were targeted) aged between 6 and 11 years. A randomized controlled trial was conducted based on a non-immersive virtual reality system where virtual objects could be managed by children in a natural way using their hands. Two different groups (i.e., experimental and control groups) were planned. A ten-week program was designed. Dependent measures included self-regulation and the acceptance of the technology. Colombini et al. (2021) generated and examined the LEAP Motion technology. The LEAP Motion controller represented a higher affordable USB capture device capable of detecting and recording, through a virtual reality-based system, natural interactions with digital contents, via an optic tracking of both hand and finger movements. NDD and neurodegenerative diseases (i.e., ADHD, ASD, mild cognitive impairments, and dementia) were targeted.

## Wearable Technologies

WT represent a basic option and play a primary role in daily life and in the healthcare industry. Covid-19 pandemic suddenly interrupted the healthcare services and negatively affected medical systems. Actually, the provision and diffusion of the vaccine progressively improved our health conditions and social interactions. However, WT have the potential to support and provide safety and assistance to both healthcare professionals and users. For instance, communication with distant partners, real-time remote monitoring, symptoms predictions, assessment, and recovery objectives may be easily pursued (Lancioni et al., 2020; Aoyagi et al., 2021; Atashzar et al., 2021; Michelin et al., 2021; Ueafuea et al., 2021).

Billeci et al. (2018) evaluated the feasibility of using WT for monitoring and recording of autonomic activity in toddlers with ASD during joint attention stimuli presentation. Twenty ASD toddlers and 20 age- and gender-matched typically developed children were recruited and observed at baseline, and during Joint attention task through non-intrusive chest strap for electrocardiography (ECG). Results showed the feasibility and effectiveness of WT to characterize the autonomic activity in ASD toddlers. Sgandurra et al. (2018) created a new medical device to provide an early, intensive, and customized intervention carried out at home by parents of infants with congenital brain damage but managed remotely by expert clinicians. A new protocol including a sample of infants at high risk of cerebral palsy was proposed through a randomized controlled trial finalized at systematically comparing a technological system to a massage intervention. Forty-two infants were enrolled. An 8-wesek program was planned with three data points, at the baseline, at the end of the intervention, and at two and 18 month-follow-up, respectively.

Di Palma et al. (2017) conducted a study, WT-based, to acquire signals during therapeutic sessions supported by interactive serious games and to correlate the autonomic activity to the engagement of the child during socio-cognitive tasks for an assessment of the intervention effect and for the customization of the therapy. Five ASD-high functioning children aged between 6 and 8 years were recruited. A wearable chest for ECG was used. A longitudinal observation along six months was recorded. Data emphasized the feasibility and the suitability of the device to be applied in clinical settings. Gualniera et al. (2021) used a web-based technology to measure the phenotype and non-invasive WT to evaluate emotional-behavioral autonomic dysregulation (EBAD) through electrodermal activity (EDA) and heart rate variability (HRV) in ten girls with Rett syndrome (RTT), after different pharmacological treatments. Results demonstrated the suitability and the effectiveness of the adopted technology. Airaksinen et al. (2020) described the implementation of an infant WT in 22 infants of seven months of age at risk of ASD and cerebral palsy to monitor their posture and movements. The technology was sensitive to the infants' movements and postures.

## Discussion

Data of the reviewed studies on both technological issues (i.e., VR and WT) evidenced the affordability, effectiveness, and suitability of the adopted technologies to ensure children and adolescents with NDD with adaptive skills. Results were largely satisfactory although along the contribution of Dellazizzo et al. (2020) a low evidence quality emerged. Cognitive and executive functions, communication and emotional competences, posture and motor movements were broadly targeted in individuals with ADHD and ASD. Conversely, participants with cerebral palsy and rare genetic diseases (e.g., Rett syndrome) were still uninvestigated as well as request and choice opportunities, leisure options, and challenging behaviors considered as a primary or a secondary outcome (Lancioni et al., 2007, 2008; Chiapparino et al., 2011; Stasolla et al., 2017; Perilli et al., 2019). The contribution of Colombini et al. (2021) combined technological solutions for NDD, neurodegenerative diseases and acquired brain injuries.

Recently, Stasolla et al. (2021) developed new virtual reality-aided solutions to remotely evaluate and recovery cognitive functioning among individuals with neurodegenerative diseases to promote cognitive empowerment (Fabio et al., 2020). Internet access and leisure option were embedded in patients with neurological disorders. Additionally, Bernini et al. (2021) implemented HomeCoRe (i.e., Home Cognitive Rehabilitative software) as an innovative approach and a valid strategy for home-based cognitive rehabilitation in neurodegenerative impairments in clinical settings, specifically useful in the Covid-19 pandemic era. Finally, Bottiroli et al. (2021) developed *Smart Aging*, a serious game platform that generates a 3D VR environment in which users may perform a set of screening tasks finalized at a broadly and global cognition's evaluation. A suitable extension might be proposed for children and adolescents with NDD. For example, one may envisage an adapted version to evaluate request, choice, and leisure opportunities and internet access. Similarly, challenging behaviors and positive participation could be recorded as primary and secondary outcomes, respectively (Stasolla et al., 2014, 2015). Alternatively, telerehabilitation strategies can be implemented or combined to remotely supervise and recovery individuals with NDD (Iannizzotto et al., 2020).

Despite the encouraging and promising perspectives, some limitations should be claimed. First, the current opinion paper was based only on ten contributions (i.e., including empirical studies, literature reviews, and protocol studies). Second, only VR and WT options were considered. Third, only children and adolescents were targeted. Fourth, only last five years range interval was embedded. In light of the above, further research on the effects of new technologies to promote adaptive skills in children and adolescents with NDD and to reduce both families and caregivers' burden can be considered highly warranted. Both assessment and rehabilitation purposes should be considered. Telemedicine combined with VR can be included as a suitable issue within this framework for individuals with neurological disorders (Matamala-Gomez et al., 2021).

Accordingly, new research perspectives should deal with the following topics: (a) participants, behaviors, and technology extensions, (b) follow-up, maintenance, preference checks, and generalization phases, and (c) social validation procedures involving external raters with a personal or a professional experience (Stasolla et al., 2019).

## Author Contributions

The author confirms being the sole contributor of this work and has approved it for publication.

## Conflict of Interest

The author declares that the research was conducted in the absence of any commercial or financial relationships that could be construed as a potential conflict of interest.
